# Different biologics for biological-naïve patients with psoriatic arthritis: a systematic review and network meta-analysis

**DOI:** 10.3389/fphar.2024.1279525

**Published:** 2024-03-13

**Authors:** Jixia Lin, Yougang Ren

**Affiliations:** ^1^ Department of Rheumatism and Immunology, Ningbo No.6 Hospital, Ningbo, Zhejiang, China; ^2^ Department of Dermatology, Ningbo No.6 Hospital, Ningbo, Zhejiang, China

**Keywords:** biologics, biological-naïve, psoriatic arthritis, efficacy, safety, network meta-analysis

## Abstract

**Aim:** To systematically compare the efficacy and safety of biologics [tumor necrosis factor inhibitors (TNFi), interleukin (IL) inhibitors, phosphodiesterase-4 inhibitors (PDE4i), and Janus kinase inhibitors (JAKi)] for biological-naïve patients with psoriatic arthritis (PsA).

**Methods:** PubMed, Web of Science, Embase, and Cochrane Library were comprehensively searched until 12 March 2023. Only head-to-head active comparison studies were included, and placebo-controlled studies without active biologic comparators were excluded. Outcomes included musculoskeletal endpoint [American College of Rheumatology (ACR) 20/50/70, resolution of enthesitis, resolution of dactylitis], function endpoint [Health Assessment Questionnaire-Disability Index (HAQ-DI) change, ∆ HAQ-DI ≥ 0.35], composite index endpoint [ACR 50 + Psoriasis Area Severity Index (PASI) 100], and adverse events. The Jadad scale and Newcastle-Ottawa scale (NOS) were adopted to evaluate the quality of eligible studies.

**Results:** Totally 17 studies with head-to-head comparisons of these biologics were included in this systematic review and network meta-analysis. Compared with IL-17A inhibitors (IL-17Ai), TNFi were associated with a lower rate of achieving ACR 20 response [pooled risk ratios (RR) = 0.92, 95% credibility interval (CrI): 0.86, 0.98]. JAKi had the greatest possibility of achieving ACR 20 (50.25%) and ACR 50 (83.03%). The JAKi group had a higher rate of achieving ACR 70 response than the IL-17Ai group (pooled RR = 1.25, 95%CrI: 1.00, 1.57); TNFi were less effective than JAKi in terms of ACR 70 (pooled RR = 0.77, 95%CrI: 0.64, 0.94). ACR 70 was most likely to be achieved in patients using JAKi (97.48%). The IL-17Ai group had a higher rate of enthesitis resolution than the TNFi group [pooled RR = 1.22, 95% confidence interval (CI): 1.02, 1.47]. Compared with IL-17Ai, TNFi were associated with a lower rate of enthesitis resolution (pooled RR = 0.80, 95%CrI: 0.72, 0.88). Patients receiving IL-17Ai had the highest likelihood of achieving enthesitis resolution (82.76%), dactylitis resolution (58.66%) and the greatest HAQ-DI change (59.74%). IL-17Ai had a similar impact in achieving ∆ HAQ-DI ≥ 0.35 to TNFi (pooled RR = 1.15, 95%CI: 0.93, 1.41). Individuals receiving IL-17Ai had a higher rate of achieving combined ACR 50 and PASI 100 response than those receiving TNFi (pooled RR = 1.56, 95%CI: 1.29, 1.88). Patients receiving PDE4i were least likely to have adverse events (41.59%).

**Conclusion:** In 2023, considering both efficacy and safety, IL-17Ai may be the better treatment option for biological-naïve patients with PsA requiring biological therapy.

## Introduction

Psoriatic arthritis (PsA) is a chronic inflammatory disorder characterized by joint and enthesis inflammation, and influences multiple organ systems, including peripheral and axial joints, entheses, skin, and nails (Ocampo and Gladman, 2019; López-Ferrer et al., 2022). This disease develops in approximately 30% of patients with psoriasis, and is a primary comorbidity of psoriasis (Gottlieb and Merola, 2020). An important concern for PsA patients is pain relief, as well as their ability to engage in social activities, fatigue, and psychological distress (Gudu and Gossec, 2018). PsA is related to decreased quality of life and a great economic burden (D'Angiolella et al., 2018; Kishimoto et al., 2021).

Nowadays, biologic disease-modifying antirheumatic drugs (bDMARDs) have been applied in the treatment of PsA due to reported efficacy and safety (Kamata and Tada, 2020; Ruyssen-Witrand et al., 2020). The main types of biological agents include tumor necrosis factor inhibitors (TNFi) (e.g., adalimumab), interleukin (IL) inhibitors (e.g., ixekizumab), Janus kinase inhibitors (JAKi) (e.g., upadacitinib), and phosphodiesterase-4 inhibitors (PDE4i) (e.g., apremilast) (Raychaudhuri et al., 2017; Singh et al., 2019). Several studies were conducted to explore the role of biologics in PsA. For example, Kristensen et al. (2022) found that compared with adalimumab, ixekizumab was more effective in achieving American College of Rheumatology (ACR) 50 and Psoriasis Area Severity Index (PASI) 100 simultaneously. According to another study, IL-17A inhibitors (IL-17Ai) exhibited similar impacts on the health assessment questionnaire (HAQ) score and ACR 20 achievement to TNFi in PsA (Izumiyama et al., 2021). For biological-naïve PsA patients, secukinumab appeared to be associated with greater ACR 20/50 response than infliximab in the medium to long term (Strand et al., 2019). Based on the existing direct and indirect evidence, increasing network meta-analyses have investigated the efficacy and safety of specific biologics (e.g., adalimumab, ustekinumab, apremilast) in PsA (Migliore et al., 2021). Nevertheless, comprehensive comparisons among broad categories of biologics, such as TNFi, IL inhibitors and JAKi, for biological-naïve patients with PsA are lacking. Besides, more studies on biologics for PsA patients are carried out in recent years. Updated network meta-analyses are needed to provide a reference for biologics selection in PsA treatment.

This latest study aimed to systematically evaluate and compare the efficacy and safety of biologics [TNFi, IL inhibitors, PDE4i, and JAKi] for biological-naïve patients with PsA via a systematic review and network meta-analysis.

## Methods

### Search strategy

PubMed, Web of Science, Embase, and Cochrane Library were comprehensively searched by two independent investigators (JX Lin, YG Ren) from inception to 12 March 2023. Disagreements were addressed via discussion. English search terms included “biological agent” OR “abatacept” OR “adalimumab” OR “apremilast” OR “certolizumab” OR “etanercept” OR “golimumab” OR “Infliximab” OR “Ixekizumab” OR “Secukinumab” OR “Tofacitinib” OR “Ustekinumab” OR “apremilast” OR “Tumor Necrosis Factor-alpha” OR “Monoclonal Antibodies” OR “Phosphodiesterase 4 Inhibitors) AND (Arthritis, Psoriatic” OR “Psoriasis, Arthritic” OR “Arthritic Psoriasis” OR “Psoriatic Arthritis” OR “Psoriasis Arthropathica” OR “Psoriatic Arthropathy” OR “Arthropathies, Psoriatic” OR “Arthropathy, Psoriatic” OR “Psoriatic Arthropathies”. Preliminary screening was conducted based on the titles and abstracts of the retrieved studies, followed by subsequent screening via full texts. This systematic review and network meta-analysis was performed according to the Preferred Reporting Items for Systematic Reviews and Meta-Analysis (PRISMA) extension statement for network meta-analyses (Hutton et al., 2015).

### Eligibility criteria

Inclusion criteria: 1) studies on biological-naïve patients with PsA (population); 2) studies on TNFi (adalimumab, golimumab, certolizumab, infliximab, etanercept), IL-17Ai (ixekizumab, secukinumab, bimekizumab) and IL-12/23 inhibitors (IL-12/23i) (ustekinumab), PDE4i (apremilast), and JAKi (upadacitinib) biologics (intervention and comparator); 3) head-to-head active comparison studies; 4) studies on any of the following outcomes: musculoskeletal endpoint [ACR 20/50/70, resolution of enthesitis evaluated by the Spondyloarthritis Research Consortium of Canada Enthesitis Index (SPARCC) and Leeds Enthesitis Index (LEI), resolution of dactylitis evaluated by the Leeds Dactylitis Index-Basic (LDI-B)], function endpoint [Health Assessment Questionnaire-Disability Index (HAQ-DI) change (pre-treatment HAQ-DI minus post-treatment HAQ-DI), ∆ HAQ-DI ≥ 0.35], composite index endpoint (ACR 50 + PASI 100), adverse events, arthritis activity endpoint [disease activity score (DAS)], skin endpoint (PASI 90, PASI 100, PASI), and drug retention (outcome); 5) randomized controlled trials (RCTs) and cohort studies (study design). Adverse events included infections, injection site reactions, malignancies, cerebrocardiovascular events, allergic reactions/hypersensitivity, inflammatory bowel disease, depression, hepatic laboratory changes, cytopenia, and neutropenia.

Exclusion criteria: 1) animal trials; 2) studies on patients who had previously received treatment with relevant biologics, or on a mixed population of biological-naïve and biological-experienced patients; 3) studies without a control group or placebo-controlled studies without active biologic comparators; 4) studies of which data were incomplete or could not be extracted; 5) case reports, meeting abstracts, protocols, letters, reviews, meta-analysis; 6) non-English literature.

### Data extraction and quality assessment

Extracted data from qualified studies included first author, year of publication, country, study design, group, sample size, age (years), sex (male/female), duration of PsA, PASI, DAS28, HAQ-DI, comorbidity, concomitant conventional synthetic disease-modifying antirheumatic drugs (csDMARDs), concomitant nonsteroidal anti-inflammatory drugs (NSAIDs), concomitant glucocorticoids, follow-up time (months), and outcomes. Two investigators (JX Lin, YG Ren) collected the above data independently.

The Jadad scale was adopted to evaluate the quality of RCTs from four dimensions: randomization, concealment of allocation, double blinding, and withdrawals and dropouts. This scale had a total score of 7, with 1–3 as low quality and 4–7 as high quality (Jadad et al., 1996). For the quality assessment of cohort studies, the Newcastle-Ottawa scale (NOS) was employed and measured three sections: population selection, intergroup comparability, and outcome measurement. The NOS had a total score of 9, with 0–3 as poor quality, 4–6 as fair quality, and 7–9 as good quality (Wells et al., 2000).

### Statistical analysis

Through constructing a Bayesian framework and a Monte Carlo Markov Chain (MCMC), this network meta-analysis was performed, with the number of model chains of 4, the number of initial iterations of 20000, the number of updated iterations of 50000, and the step size of 1. Heterogeneity indicated the overall degree of difference in the same pair of comparisons. The I^2^ statistic was the primary indicator of statistical heterogeneity, with values < 25%, 25%–50% and >50% representing low, moderate and high heterogeneity, separately. Network plots were depicted to show direct and indirect comparisons of biologics for each outcome. A larger node indicated a larger sample size for the biologics represented by the node, while a thicker line indicates a larger number of studies for the comparison of biologics at both ends of the line. The influences of biologics on the outcomes were illustrated via forest plots and league tables. Rank probabilities exhibited the probability of different biologics ranking at a certain position (e.g., ranking first, second, third). For HAQ-DI change, weighted mean differences (WMDs) and 95% credibility intervals (CrIs) were described for different biological agents; for ACR 20/50/70, resolution of enthesitis, resolution of dactylitis, and adverse events, risk ratios (RRs) and 95%CrIs were estimated. Statistical analysis was completed by applying STATA 15.1 (Stata Corporation, College Station, TX, United States) and R 4.1.3 (R Foundation for Statistical Computing, Vienna, Austria).

## Results

### Characteristics of the included studies

**FIGURE 1:**
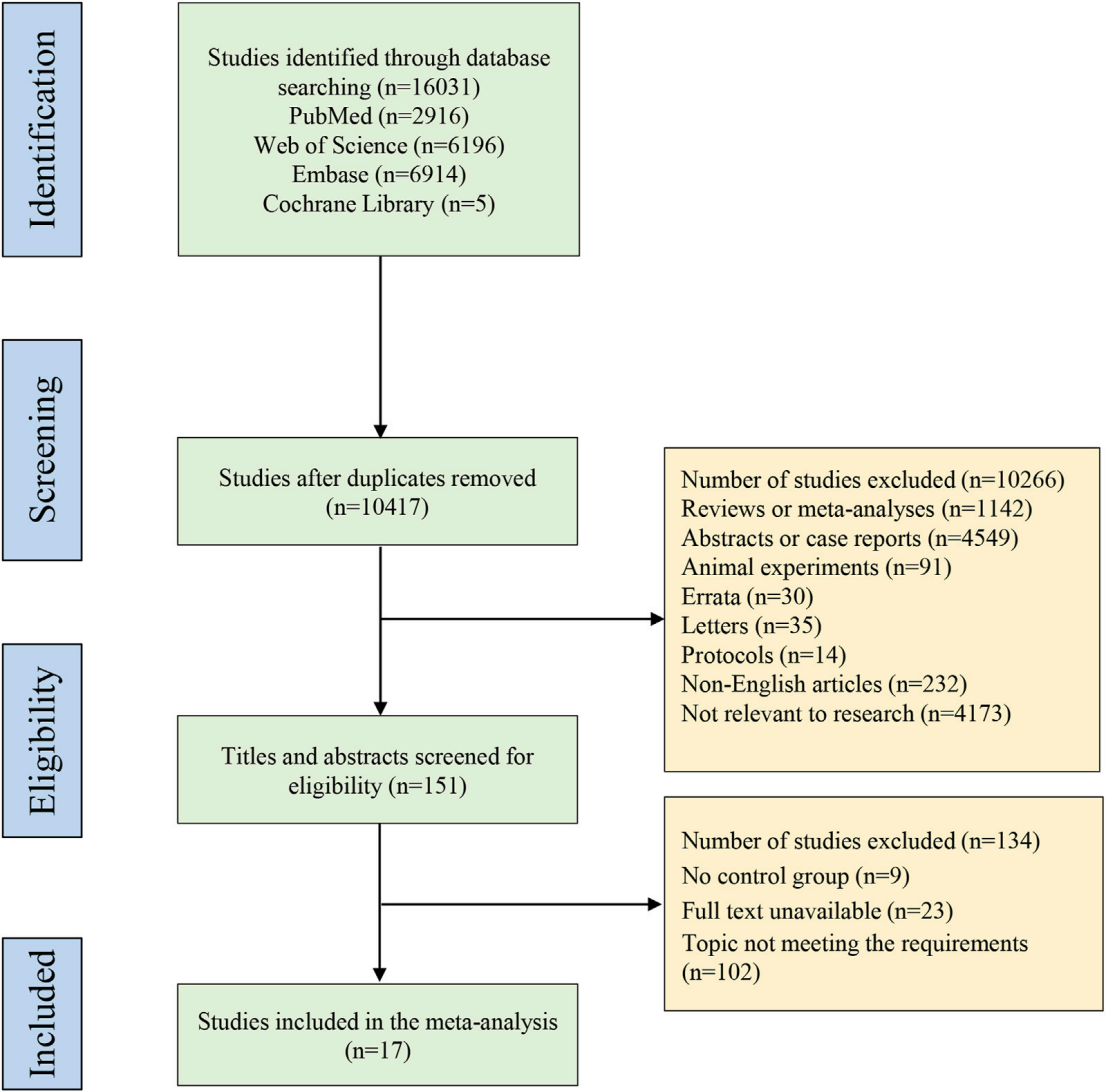
PRISMA flow diagram of study selection. PRISMA, Preferred Reporting Items for Systematic Reviews and Meta-Analysis.

A total of 16,031 studies were identified through database searching, with 2,916 from PubMed, 6,196 from Web of Science, 6,914 from Embase, and 5 from Cochrane Library. Subsequently, 10,417 studies left after duplicate removal, followed by screening with titles and abstracts, and then full texts based on the eligibility criteria. Ultimately, 17 studies (Atteno et al., 2010; Araujo et al., 2019; Strand et al., 2019; McInnes et al., 2020; Mease et al., 2020; Smolen et al., 2020; Gottlieb et al., 2021; Izumiyama et al., 2021; Lindström et al., 2021; McInnes et al., 2021; Eviatar et al., 2022; Kristensen et al., 2022; McInnes et al., 2022; Pina Vegas et al., 2022; Reich et al., 2022; Silva et al., 2022; McInnes et al., 2023) including head-to-head comparisons of the biologics were included in this systematic review and network meta-analysis. Figure 1 presents the detailed process of study selection. These included studies were published from 2010 to 2023. Most of the studies came from the United States. Ten studies (Araujo et al., 2019; McInnes et al., 2020; Mease et al., 2020; Smolen et al., 2020; Gottlieb et al., 2021; McInnes et al., 2021; Kristensen et al., 2022; McInnes et al., 2022; Reich et al., 2022; McInnes et al., 2023) were RCTs, of which 2 (Mease et al., 2020; Reich et al., 2022) had low quality, and 8 (Araujo et al., 2019; McInnes et al., 2020; Smolen et al., 2020; Gottlieb et al., 2021; McInnes et al., 2021; Kristensen et al., 2022; McInnes et al., 2022; McInnes et al., 2023) had high quality; 7 (Atteno et al., 2010; Strand et al., 2019; Izumiyama et al., 2021; Lindström et al., 2021; Eviatar et al., 2022; Pina Vegas et al., 2022; Silva et al., 2022) studies were cohort studies, with 6 (Atteno et al., 2010; Izumiyama et al., 2021; Lindström et al., 2021; Eviatar et al., 2022; Pina Vegas et al., 2022; Silva et al., 2022) of fair quality, and 1 (Strand et al., 2019) of good quality. Characteristics and quality assessment of the included studies are illustrated in Tables 1, 2, respectively.

**TABLE 1:** Characteristics of the included studies.

Author	Year	Country	Study design	Group	Sample size	Age, years	Sex (male/female), n	Duration of PsA, years	Basic PASI	Basic DAS28	Basic HAQ-DI	Comorbidity, n	Concomitant csDMARDs, n	Concomitant NSAIDs, n	Concomitant glucocorticoids, n	Follow up, months	Outcomes measured
Araujo	2018	Germany	RCT	Ustekinumab	23	62 ± 18	10/13	2 ± 6.0	3.0 ± 6.6	4.0 ± 1.09	0.87 ± 0.63	NA	MTX, 19	NA	0	6	DAS28, PASI 90, PASI 100, HAQ-DI
				TNFi	24	58 ± 21	18/6	3 ± 4.8	2.8 ± 3.6	4.4 ± 1.24	1.17 ± 0.62	NA	MTX, 24	NA	1		
Atteno	2010	Italy	Cohort study	Etanercept	36	49.3 ± 13.4	15/21	80 (20–140) m	26 ± 18.5	NA	1.2 ± 0.4	NA	NA	NA	NA	12	PASI, HAQ-DI
				Adalimumab	34	47.5 ± 11.5	14/20		18 ± 16.5	NA	1.2 ± 0.3	NA	NA	NA	NA		
				Infliximab	30	48.5 ± 12.9	11/19		15 ± 14.8	NA	1.5 ± 0.5	NA	NA	NA	NA		
da Silva	2022	Brazil	Cohort study	Adalimumab	91	50.92 ± 11.89	40/51	5.36 ± 7.27	NA	NA	1.23 ± 0.74	68	43	25	27	12	HAQ, EQ-5D
				Etanercept	52	51.50 ± 12.90	19/33	4.61 ± 6.25	NA	NA	1.21 ± 0.71	40	15	9	9		
Eviatar	2022	Israel	Cohort study	Secukinumab	13	41.2 ± 14.4	NA	13.7 ± 13.1	NA	NA	NA	NA	NA	NA	NA	12	Drug retention
				Etanercept	130	42.6 ± 14.5	NA	7.8 ± 8.6	NA	NA	NA	NA	NA	NA	NA		
				Infliximab	28	41.2 ± 4.2	NA	8.5 ± 9.4	NA	NA	NA	NA	NA	NA	NA		
				Adalimumab	103	41.5 ± 14.0	NA	8.7 ± 9.0	NA	NA	NA	NA	NA	NA	NA		
				Golimumab	25	39.9 ± 15.7	NA	12.1 ± 11.3	NA	NA	NA	NA	NA	NA	NA		
Gottlieb	2021	United States	RCT	Secukinumab	110	48.9 ± 12.2	66/44	6.1 ± 8.9	16.2 ± 9.6	4.7 ± 0.9	1.3 ± 0.6	NA	Included but not limited to MTX	Leflunomide	Prednisone, 8	13	ACR 20/50/70, ACR 50 + PASI 100, HAQ-DI change, HAQ-DI ≥ 0.35, resolution of enthesitis, resolution of dactylitis
				Adalimumab	101	46.9 ± 12.3	57/44	6.7 ± 8.4	15.0 ± 8.9	4.8 ± 1.0	1.3 ± 0.7	NA			Prednisone, 5		
Izumiyama	2021	Japan	Cohort study	TNFi	13	44.3 ± 9.5	9/4	57.7 ± 57.7 m)	NA	3.1 ± 1.1	0.26 ± 0.44	NA	MTX, 10	NA	NA	13	HAQ-DI change, ACR20
				IL-17Ai	18	55.2 ± 9.2	9/9	85.6 ± 67.7 m)	NA	3.0 ± 1.9	0.47 ± 0.46	NA	MTX, 7	NA	NA		
Kristensen	2022	Denmark	RCT	Ixekizumab	234	48.0 ± 12.1	132/102	6.5 ± 7.4	4.7 ± 3.5	NA	1.2 ± 0.6	NA	MTX, 47	NA	47	13	HAQ-DI ≥ 0.35, resolution of enthesitis, resolution of dactylitis
				Adalimumab	231	48.7 ± 12.5	116/115	5.9 ± 6.4	4.9 ± 2.9	NA	1.2 ± 0.7	NA	MTX, 42	NA	42		
Lindstrom	2021	Sweden	Cohort study	Adalimumab	579	48 ± 13	300/279	10 ± 10	NA	27 ± 17	NA	30	MTX, 13; Sulphasalazine, 3	NA	NA	6	Drug retention, adverse event
				Secukinumab	165	52 ± 13	79/86	12 ± 10	NA	30 ± 17	NA	66	MTX, 5; Sulphasalazine, 2	NA	NA		
				Etanercept	1786	49 ± 13	811/975	10 ± 10	NA	26 ± 16	NA	36	MTX, 7; Sulphasalazine, 2	NA	NA		
				Infliximab	1,006	49 ± 13	464/542	10 ± 10	NA	27 ± 16	NA	33	MTX, 35; Sulphasalazine, 7	NA	NA		
				Golimumab	215	46 ± 13	103/112	9 ± 7	NA	24 ± 13	NA	38	MTX, 10; Sulphasalazine, 4	NA	NA		
				Certolizumab	272	47 ± 13	124/148	10 ± 9	NA	29 ± 14	NA	27	MTX, 33; Sulphasalazine, 9	NA	NA		
McInnes	2020	United States	RCT	Secukinumab	426	48.5 ± 12.38	208/218	5.1 ± 7.60	10.6 ± 9.00	4.7 ± 1.00	1.3 ± 0.64	NA	NA	NA	61	13	ACR 20/50/70, HAQ-DI change, ACR 50 + PASI 100, HAQ-DI ≥0.35, adverse event, resolution of enthesitis, resolution of dactylitis
				Adalimumab	427	49.5 ± 12.44	229/198	5.7 ± 7.29	10.0 ± 8.15	4.7 ± 0.94	1.2 ± 0.64	NA	NA	NA	58		
McInnes	2021	United States	RCT	Upadacitinib	429	51.6 ± 12.2	191/238	6.2 ± 7.4	9.8 ± 10.0	NA	1.2 ± 0.7	NA	353	NA	73	6	ACR20, adverse event, resolution of enthesitis, resolution of dactylitis
				Adalimumab	429	51.4 ± 12.0	207/222	5.9 ± 7.1	9.4 ± 8.5	NA	1.1 ± 0.6	NA	347	NA	72		
McInnes	2022	United States	RCT	Upadacitinib	429	51.6 ± 12.2	191/238	6.2 ± 7.4	9.8 ± 10.0	NA	1.2 ± 0.7	NA	353	NA	73	24	ACR 20/50/70
				Adalimumab	429	51.4 ± 12.0	207/222	5.9 ± 7.1	9.4 ± 8.5	NA	1.1 ± 0.6	NA	347	NA	72		
McInnes	2023	United Kingdom	RCT	Bimekizumab	431	48.5 ± 12.6	201/230	6.0 ± 7.3	8.2 ± 6.8	NA	0.82 ± 0.59	NA	301	NA	NA	6	ACR 20/50/70, HAQ-DI change, HAQ-DI ≥0.35, resolution of enthesitis, resolution of dactylitis, adverse event
				Adalimumab	140	49.0 ± 12.8	71/69	6.1 ± 6.8	8.5 ± 7.6	NA	0.86 ± 0.54	NA	99	NA	NA		
Mease	2020	United States	RCT	Ixekizumab	283	47.5 ± 12.0	162/121	6.6 ± 7.4	7.9 ± 8.7	5.8 ± 0.9	1.2 ± 0.6	NA	193	NA	NA	6	ACR 50+PASI 100, ACR 20/50/70, HAQ-DI ≥0.35
				Adalimumab	283	48.3 ± 12.3	150/133	5.9 ± 6.4	7.7 ± 7.3	5.8 ± 1.0	1.3 ± 0.7	NA	199	NA	NA		
Reich	2022	United States	RCT	Ixekizumab	49	45.3 ± 11.5	30/19	7.0 ± 7.4	22.9 ± 10.5	NA	NA	NA	MTX, 25	NA	NA	13	ACR 20/50/70, adverse event
				Adalimumab	51	46.3 ± 11.3	33/18	5.7 ± 6.2	20.5 ± 7.3	NA	NA	NA	MTX, 28	NA	NA		
Smolen	2020	Austria	RCT	Ixekizumab	283	47.5 ± 12.0	162/121	6.6 ± 7.4	7.9 ± 8.7	5.8 ± 0.9	1.2 ± 0.6	NA	193	NA	NA	13	ACR 50+PASI 100, ACR 20/50/70, HAQ-DI ≥0.35, adverse event, resolution of enthesitis, resolution of dactylitis
				Adalimumab	283	48.3 ± 12.3	150/133	5.9 ± 6.4	7.7 ± 7.3	5.8 ± 1.0	1.3 ± 0.7	NA	199	NA	NA		
Strand	2019	United States	Cohort study	Secukinumab	238	48.6 ± 11.8	116/122	NA	11.4 ± 12.7	NA	1.1 ± 0.6	83	MTX, 47	NA	NA	12	ACR 20/50/70
				Infliximab	100	47.1 ± 12.8	71/29	NA	12.2 ± 11.3	NA	1.2 ± 0.6	126	MTX, 105	NA	NA		
Vegas	2022	France	Cohort study	TNFi	7,289	48.2 ± 12.8	3,002/4,287	NA	NA	NA	NA	3,150	2,992	1,473	NA	12 (6–25)	MACE
				IL-12/23i	1,058	49.8 ± 12.8	475/583	NA	NA	NA	NA	708	305	144	NA		
				IL-17Ai	1,163	49.2 ± 12.2	482/681	NA	NA	NA	NA	639	336	188	NA		
				Apremilast	1885	54.0 ± 12.5	835/1,050	NA	NA	NA	NA	1,175	653	357	NA		

RCT, randomized controlled trials; PsA, psoriatic arthritis; PASI, psoriasis area severity index; DAS28, disease activity score 28; HAQ-DI, health assessment questionnaire disability index; MTX, methotrexate; csDMARDs, conventional synthetic disease-modifying antirheumatic drugs; NSAIDs, nonsteroidal anti-inflammatory drugs; TNFi, tumor necrosis factor inhibitors; IL-17Ai, interleukin-17A, inhibitors; EQ-5D, european quality of life five dimensions; MACE, major adverse cardiac event; ACR, american college of rheumatology; NA, not applicable.

**TABLE 2:** Quality assessment of the included studies.

Author	Year	Country	Study design	Randomization	Concealment of allocation	Double blinding	Withdrawals and dropouts	Total score
Araujo	2018	Germany	RCT	1	2	1	1	5
Gottlieb	2021	United States	RCT	1	2	1	0	4
Kristensen	2022	Denmark	RCT	2	2	1	1	6
McInnes	2020	United States	RCT	1	2	1	0	4
McInnes	2021	United States	RCT	1	2	0	1	4
McInnes	2022	United States	RCT	2	1	0	1	4
McInnes	2023	United Kingdom	RCT	2	2	1	1	6
Mease	2020	United States	RCT	1	1	1	0	3
Reich	2022	United States	RCT	2	0	0	0	2
Smolen	2020	Austria	RCT	1	2	0	1	4
Author	Year	Country	Study design	Selection	Comparability	Outcome	Total score	
Atteno	2010	Italy	Cohort study	⟷⟷	⟷	⟷⟷	5	
da Silva	2022	Brazil	Cohort study	⟷⟷	⟷	⟷⟷	5	
Eviatar	2022	Israel	Cohort study	⟷⟷	⟷	⟷⟷	5	
Izumiyama	2021	Japan	Cohort study	⟷⟷	⟷	⟷⟷	5	
Lindstrom	2021	Sweden	Cohort study	⟷⟷	⟷⟷	⟷⟷	6	
Strand	2019	United States	Cohort study	⟷⟷⟷	⟷	⟷⟷⟷	7	
Vegas	2022	France	Cohort study	⟷⟷	⟷⟷	⟷⟷	6	

The Jadad scale was adopted to evaluate the quality of RCTs from four dimensions: randomization, concealment of allocation, double blinding, and withdrawals and dropouts. For the quality assessment of cohort studies, the Newcastle-Ottawa scale (NOS) was employed and measured three sections: population selection, intergroup comparability, and outcome measurement.

RCT, randomized controlled trials.

### Different biologics for the musculoskeletal endpoint

#### ACR 20

**FIGURE 2:**
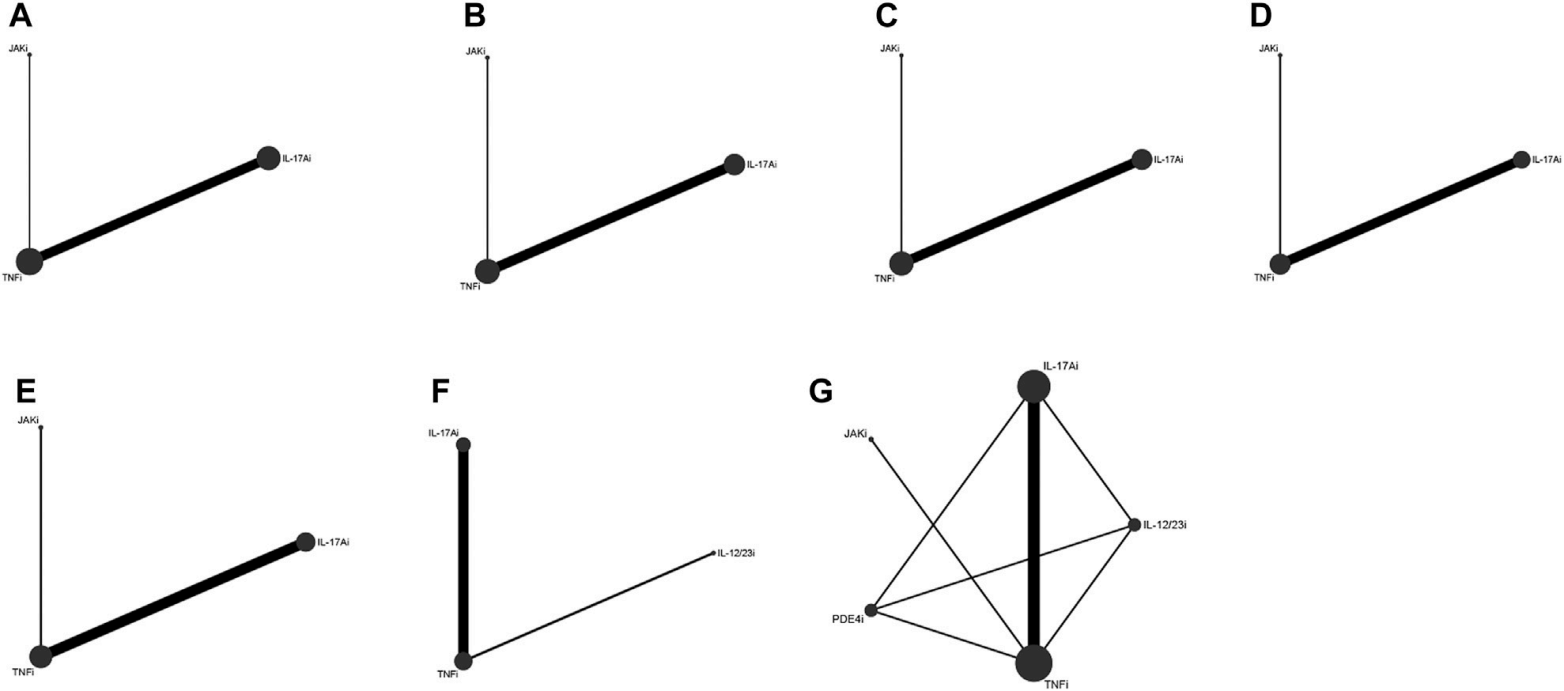
Network plots of various biologics for different outcomes in biological-naïve patients with PsA. **(A)**, ACR 20; **(B)**, ACR 50; **(C)**, ACR 70; **(D)**, resolution of enthesitis; **(E)**, resolution of dactylitis; **(F)**, HAQ-DI change; **(G)**, adverse events. PsA, psoriatic arthritis; ACR, American College of Rheumatology; HAQ-DI, Health Assessment Questionnaire-Disability Index; TNFi, tumor necrosis factor inhibitors; IL-17Ai, interleukin-17A inhibitors; IL-12/23i, interleukin-12/23 inhibitors; PDE4i, phosphodiesterase-4 inhibitors; JAKi, Janus kinase inhibitors.

**FIGURE 3:**
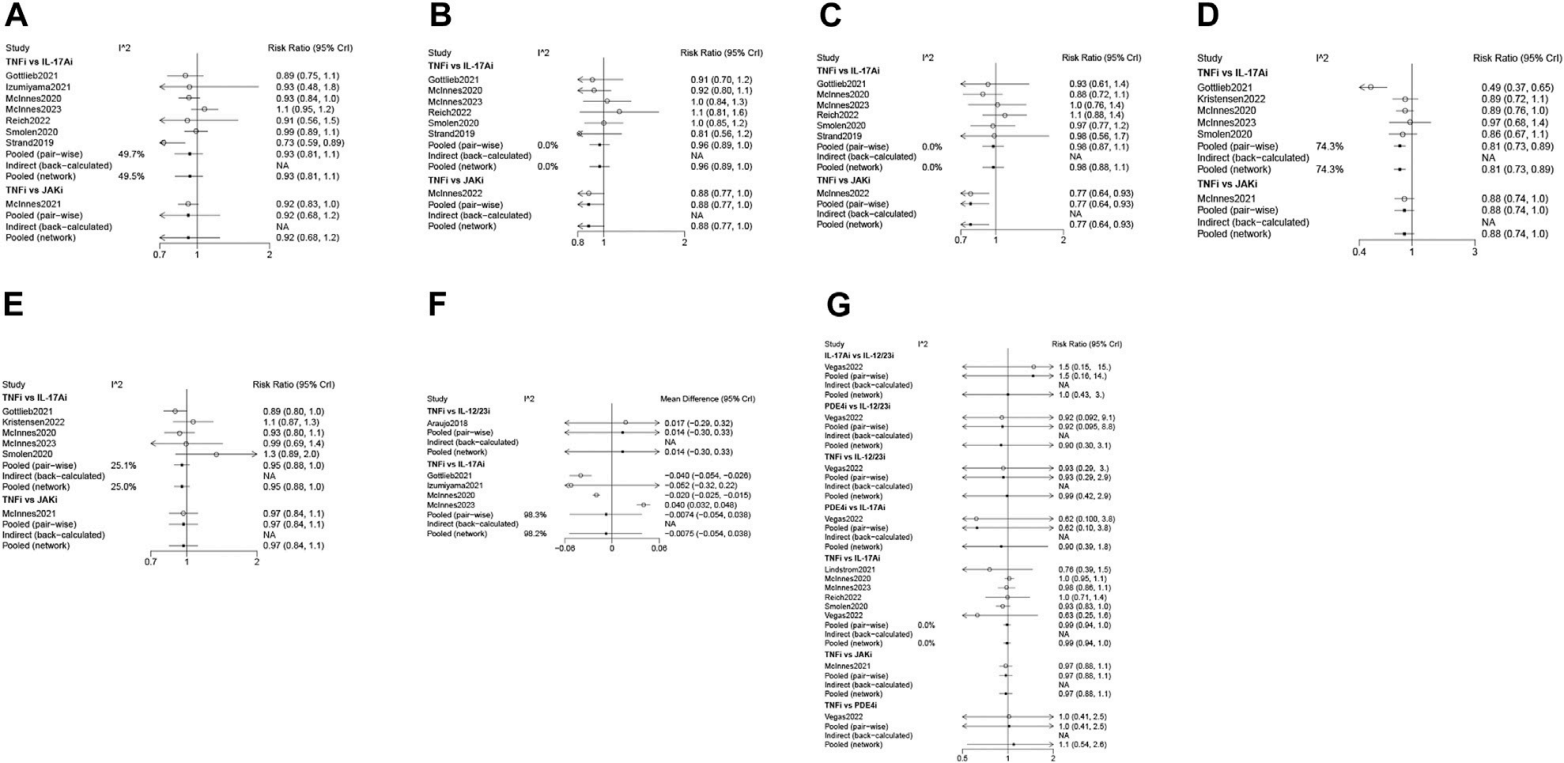
Forest plots of various biologics for different outcomes in biological-naïve patients with PsA. **(A)**, ACR 20; **(B)**, ACR 50; **(C)**, ACR 70; **(D)**, resolution of enthesitis; **(E)**, resolution of dactylitis; **(F)**, HAQ-DI change; **(G)**, adverse events. PsA, psoriatic arthritis; ACR, American College of Rheumatology; HAQ-DI, Health Assessment Questionnaire-Disability Index; TNFi, tumor necrosis factor inhibitors; IL-17Ai, interleukin-17A inhibitors; IL-12/23i, interleukin-12/23 inhibitors; PDE4i, phosphodiesterase-4 inhibitors; JAKi, Janus kinase inhibitors; CrI, credibility interval.

Eight studies (Strand et al., 2019; McInnes et al., 2020; Smolen et al., 2020; Gottlieb et al., 2021; Izumiyama et al., 2021; McInnes et al., 2021; Reich et al., 2022; McInnes et al., 2023) with 3,358 patients were eligible for ACR 20 evaluation, involving IL-17Ai, TNFi and JAKi biologics. TNFi and IL-17Ai were directly compared in more studies. TNFi were the most frequently used agent (Figure 2A). No significant differences were found in ACR 20 response among the TNFi, IL-17Ai and JAKi groups according to the forest plot (Figure 3A) and the league table (Table 3). The rank probabilities indicated that JAKi had the greatest possibility of achieving ACR 20 (68.79%), followed by IL-17Ai and TNFi (Table 4).

**TABLE 3:** League table of various biologics for different outcomes in biological-naïve patients with PsA.

ACR 20 [RR (95%CrI)]				
IL-17Ai	1.03 (0.92, 1.15)	0.95 (0.89, 1.00)		
0.97 (0.87, 1.09)	JAKi	0.92 (0.83, 1.01)		
1.06 (1.00, 1.12)	1.09 (0.99, 1.20)	TNFi		
ACR 50 [RR (95%CrI)]				
IL-17Ai	1.10 (0.94, 1.29)	0.96 (0.89, 1.05)		
0.91 (0.78, 1.07)	JAKi	0.88 (0.77, 1.00)		
1.04 (0.95, 1.13)	1.14 (1.00, 1.30)	TNFi		
ACR 70 [RR (95%CrI)]				
IL-17Ai	1.26 (1.01, 1.57)	0.98 (0.88, 1.09)		
0.79 (0.64, 0.99)	JAKi	0.77 (0.64, 0.93)		
1.02 (0.92, 1.14)	1.29 (1.07, 1.57)	TNFi		
Resolution of enthesitis [RR (95%CrI)]				
IL-17Ai	0.92 (0.76, 1.12)	0.81 (0.73, 0.89)		
1.08 (0.89, 1.32)	JAKi	0.88 (0.74, 1.04)		
1.23 (1.12, 1.36)	1.14 (0.96, 1.35)	TNFi		
Resolution of dactylitis [RR (95%CrI)]				
IL-17Ai	0.98 (0.84, 1.16)	0.95 (0.88, 1.03)		
1.02 (0.86, 1.19)	JAKi	0.97 (0.84, 1.11)		
1.05 (0.97, 1.13)	1.03 (0.90, 1.19)	TNFi		
HAQ-DI change [WMD (95%CrI)]				
IL-12/23i	0.02 (−0.30, 0.34)	0.02 (−0.30, 0.33)		
−0.02 (−0.34, 0.30)	IL-17Ai	−0.01 (−0.05, 0.04)		
0.02 (−0.33, 0.30)	0.01 (−0.04, 0.05)	TNFi		
Adverse events [RR (95%CrI)]				
IL-12/23i	1.01 (0.43, 2.96)	1.03 (0.44, 3.02)	0.91 (0.30, 3.04)	0.99 (0.43, 2.92)
0.99 (0.34, 2.31)	IL-17Ai	1.02 (0.91, 1.14)	0.90 (0.38, 1.84)	0.99 (0.94, 1.04)
0.97 (0.33, 2.28)	0.98 (0.88, 1.10)	JAKi	0.88 (0.38, 1.82)	0.97 (0.88, 1.07)
1.10 (0.33, 3.34)	1.11 (0.54, 2.60)	1.13 (0.55, 2.66)	PDE4i	1.09 (0.54, 2.57)
1.01 (0.34, 2.35)	1.01 (0.96, 1.07)	1.03 (0.94, 1.14)	0.91 (0.39, 1.86)	TNFi

Note: The data on the upper right (with the diagonal line as the dividing line) were used. These data were presented with the agent on the left as the reference group and the agent on the lower as the intervention group. For example, 1.03 (0.92, 1.15) indicated the RR and 95%CrI of the JAKi group versus the IL-17Ai group.

PsA, psoriatic arthritis; ACR, american college of rheumatology; HAQ-DI, Health Assessment Questionnaire-Disability Index; TNFi, tumor necrosis factor inhibitors; IL-17Ai, interleukin-17A inhibitors; IL-12/23i, interleukin-12/23 inhibitors; PDE4i, phosphodiesterase-4 inhibitors; JAKi, Janus kinase inhibitors; WMD, weighted mean difference; RR, risk ratio; CrI, credibility interval.

**TABLE 4:** Rank probabilities of various biologics for different outcomes in biological-naïve patients with PsA.

ACR 20					
	Ranking first	Ranking second	Ranking third		
IL-17Ai	0.310970	0.661085	0.027945		
JAKi	0.687935	0.271005	0.041060		
TNFi	0.001095	0.067910	0.930995		
ACR 50					
	Ranking first	Ranking second	Ranking third		
IL-17Ai	0.119795	0.678310	0.201895		
JAKi	0.874495	0.100485	0.025020		
TNFi	0.005710	0.221205	0.773085		
ACR 70					
	Ranking first	Ranking second	Ranking third		
IL-17Ai	0.018030	0.654235	0.327735		
JAKi	0.980615	0.016120	0.003265		
TNFi	0.001355	0.329645	0.669000		
Resolution of enthesitis (LEI = 0)					
	Ranking first	Ranking second	Ranking third		
IL-17Ai	0.788715	0.21128	0.000005		
JAKi	0.211285	0.72443	0.064285		
TNFi	0.000000	0.06429	0.935710		
Resolution of dactylitis (LDI-B = 0)					
	Ranking first	Ranking second	Ranking third		
IL-17Ai	0.554005	0.368845	0.07715		
JAKi	0.412945	0.273585	0.31347		
TNFi	0.033050	0.357570	0.60938		
HAQ-DI change					
	Ranking first	Ranking second	Ranking third		
IL-12/23i	0.40363	0.045975	0.524465		
IL-17Ai	0.596365	0.443720	0.181825		
TNFi	0.000005	0.510305	0.293710		
Adverse events					
	Ranking first	Ranking second	Ranking third	Ranking fourth	Ranking fifth
IL-12/23i	0.394440	0.091285	0.021970	0.149435	0.342870
IL-17Ai	0.110960	0.287290	0.333315	0.206310	0.062125
JAKi	0.227275	0.319630	0.251960	0.142000	0.059135
PDE4i	0.242450	0.138225	0.027840	0.173755	0.417730
TNFi	0.024875	0.163570	0.364915	0.328500	0.118140

Note: The data indicated the probability of different biologics ranking at a certain position (e.g., ranking first, second, third).

PsA, psoriatic arthritis; ACR, american college of rheumatology; HAQ-DI, Health Assessment Questionnaire-Disability Index; TNFi, tumor necrosis factor inhibitors; IL-17Ai, interleukin-17A inhibitors; IL-12/23i, interleukin-12/23 inhibitors; PDE4i, phosphodiesterase-4 inhibitors; JAKi, Janus kinase inhibitors; LEI, leeds enthesitis index; LDI-B, Leeds Dactylitis Index-Basic.

#### ACR 50

ACR 50 was measured by 7 studies (Strand et al., 2019; McInnes et al., 2020; Smolen et al., 2020; Gottlieb et al., 2021; McInnes et al., 2022; Reich et al., 2022; McInnes et al., 2023) including 3,333 patients. IL-17Ai, TNFi and JAKi biologics were compared. More studies made direct comparison between TNFi and IL-17Ai, and more patients used TNFi (Figure 2B). Based on the forest plot (Figure 3B) and league table (Table 3), patients receiving IL-17Ai, TNFi and JAKi had similar ACR 50. The rank probabilities showed that JAKi were most likely to achieve ACR 50 (87.45%), followed by IL-17Ai and TNFi (Table 4).

#### ACR 70

Seven studies (Strand et al., 2019; McInnes et al., 2020; Smolen et al., 2020; Gottlieb et al., 2021; McInnes et al., 2022; Reich et al., 2022; McInnes et al., 2023) of 3,333 patients provided data on ACR 70, and IL-17Ai, TNFi and JAKi biologics were involved. More studies directly compared TNFi and IL-17Ai. TNFi were the most commonly used agent (Figure 2C). The forest plot found that compared with JAKi, TNFi were associated with a significantly lower rate of achieving ACR 70 response (pooled RR = 0.77, 95%CrI: 0.64, 0.93) (Figure 3C). The JAKi group had a significantly higher rate of achieving ACR 70 response than the IL-17Ai group (pooled RR = 1.26, 95%CrI: 1.01, 1.57); TNFi were less effective than JAKi in terms of ACR 70 (pooled RR = 0.77, 95%CrI: 0.64, 0.93), as demonstrated by the league table (Table 3). According to the rank probabilities, ACR 70 was most likely to be achieved in patients using JAKi (97.48%), followed by those using IL-17Ai and TNFi (Table 4).

#### Resolution of enthesitis

##### SPARCC

Three studies (McInnes et al., 2020; Smolen et al., 2020; Kristensen et al., 2022) involving 1,491 patients evaluated the role of IL-17Ai and TNFi biologics for enthesitis resolution defined by SPARCC = 0. As illustrated by pooled analysis, the IL-17Ai group had a significantly higher rate of enthesitis resolution than the TNFi group (pooled RR = 1.22, 95%CI: 1.02, 1.47, *p* = 0.032).

##### LEI

Enthesitis resolution defined by LEI = 0 was assessed by 6 studies (McInnes et al., 2020; Smolen et al., 2020; Gottlieb et al., 2021; McInnes et al., 2021; Kristensen et al., 2022; McInnes et al., 2023) in 2,633 patients who were treated with IL-17Ai, TNFi and JAKi biologics. More studies directly compared TNFi and IL-17Ai, and TNFi were the most commonly applied agent (Figure 2D). The forest plot (Figure 3D) and the league table (Table 3) showed that the rate of enthesitis resolution was significantly lower in the TNFi group than that in the IL-17Ai group (pooled RR = 0.81, 95%CrI: 0.73, 0.89). From the rank probabilities, patients receiving IL-17Ai were most likely to have enthesitis resolution (78.87%), followed by those receiving JAKi and TNFi (Table 4).

#### Resolution of dactylitis

##### LDI-B

Six studies (McInnes et al., 2020; Smolen et al., 2020; Gottlieb et al., 2021; McInnes et al., 2021; Kristensen et al., 2022; McInnes et al., 2023) compared IL-17Ai, TNFi and JAKi biologics for dactylitis resolution defined by LDI-B = 0 in 1950 patients. The direct comparison of TNFi and IL-17Ai was found in more studies, and the most patients used TNFi (Figure 2E). No significant differences were found in dactylitis resolution among IL-17Ai, TNFi and JAKi, according to the forest plot (Figure 3E) and league table (Table 3). The rank probabilities suggested that IL-17Ai had the greatest likelihood to achieve the resolution of dactylitis (55.40%), followed by JAKi and TNFi (Table 4).

### Different biologics for the function endpoint

#### HAQ-DI change

Five studies (Araujo et al., 2019; McInnes et al., 2020; Gottlieb et al., 2021; Izumiyama et al., 2021; McInnes et al., 2023) with 1,535 patients were quantified for HAQ-DI change assessment, involving IL-17Ai, TNFi and IL-12/23i biologics. TNFi and IL-17Ai were directly compared in more studies. TNFi were the most frequently used agent (Figure 2F). No significant differences were observed in HAQ-DI change among IL-17Ai, TNFi and IL-12/23i according to the forest plot (Figure 3F) and league table (Table 3). The rank probabilities indicated that patients receiving IL-17Ai had the highest likelihood of achieving the greatest HAQ-DI change (59.64%), followed by those receiving TNFi and IL-12/23i (Table 4).

#### ∆ HAQ-DI ≥ 0.35

The condition of ∆ HAQ-DI ≥ 0.35 was assessed in 5 studies (McInnes et al., 2020; Smolen et al., 2020; Gottlieb et al., 2021; Kristensen et al., 2022; McInnes et al., 2023) including 2,617 patients, and IL-17Ai and TNFi biologics were compared. Pooled analysis showed that IL-17Ai had a similar impact in achieving ∆ HAQ-DI ≥ 0.35 to TNFi [pooled RR = 1.15, 95% confidence interval (CI): 0.93, 1.41, *p* = 0.194].

#### Different biologics for the composite index endpoint

##### ACR 50 + PASI 100

As for combined ACR 50 and PASI 100 response, 3 studies (McInnes et al., 2020; Smolen et al., 2020; Gottlieb et al., 2021) with 1,194 patients were included for comparison of IL-17Ai and TNFi biologics. Pooled analysis demonstrated that patients receiving IL-17Ai had a significantly higher rate of achieving combined ACR 50 and PASI 100 response than those receiving TNFi (pooled RR = 1.56, 95%CI: 1.29, 1.88, *p* < 0.001).

##### Different biologics for adverse events

Seven studies (McInnes et al., 2020; Smolen et al., 2020; Lindström et al., 2021; McInnes et al., 2021; Pina Vegas et al., 2022; Reich et al., 2022; McInnes et al., 2023) with 15,087 patients provided data on adverse events, involving IL-17Ai, TNFi, JAKi, IL-12/23i and PDE4i biologics. There were more studies directly comparing TNFi and IL-17Ai, and more patients receiving TNFi (Figure 2G). The incidences of adverse events were comparable among these biologics based on the forest plot (Figure 3G) and league table (Table 3). The rank probabilities showed that patients receiving PDE4i were least likely to have adverse events (41.77%), following by IL-17Ai, TNFi, JAKi, and IL-12/23i (Table 4).

According to the study of McInnes et al. (2021), the incidence of serious adverse events was higher in patients receiving 30-mg dose of upadacitinib versus those receiving adalimumab and 15-mg dose of upadacitinib (6.1% vs. 3.7% and 3.1%). The adalimumab group exhibited more serious adverse events than the ixekizumab group (9.8% vs. 0.0%) (Reich et al., 2022). By week 24, 17 (4%) patients in the bimekizumab group and five (4%) patients in the adalimumab group at baseline had serious adverse events (McInnes et al., 2023).

##### Different biologics for the arthritis activity endpoint

Araujo et al. (2019) randomized patients to receive IL-12/23i and TNFi in a 1:1 ratio. After 24 weeks of follow-up, DAS28 scores were compared between the IL-12/23i and TNFi groups, and no statistical difference was found between the two groups (*p* > 0.05).

##### Different biologics for the skin endpoint

According to the study of Araujo et al. (2019), PASI 100 was found in 59% of patients treated with ustekinumab and 29% of those treated with TNFi (*p* = 0.039), while 86% of patients receiving ustekinumab and 29% of patients receiving TNFi showed PASI 90 (*p* = 0.0001). Atteno et al. (2010) compared the efficacy of three kinds of TNFi (etanercept, adalimumab, and infliximab), and the difference in PASI was statistically significant at 1 year of follow-up (*p* < 0.01). Compared with patients receiving etanercept, those receiving adalimumab (*p* < 0.01) and infliximab (*p* < 0.001) showed greater improvement in the expansion of psoriasis rash.

##### Different biologics for drug retention

Eviatar et al. (2022) reported that as a first-line treatment, the drug retention of secukinumab (IL-17Ai) was similar to that of other TNFi, except for the poor drug retention of golimumab. Three years later, 76% of patients still used secukinumab, while 50% of patients used etanercept, 52% used infliximab, 56% used adalimumab, and 34% used golimumab. Lindström et al. (2021) showed that the 1-year treatment retention rates of secukinumab and adalimumab were similar. However, there were some differences between different TNFi, and the retention rates of infliximab and certolizumab pegol showed a decreasing trend.

## Discussion

To the best of our knowledge, the current network meta-analysis was the first to compare and rank TNFi, IL-17Ai, IL-12/23i, PDE4i, and JAKi biologics based on their efficacy and safety in biological-naïve patients with PsA. With 16 studies included, it was found that JAKi had the highest probability of achieving ACR 20/50/70 response, IL-17Ai were most likely to realize the resolution of enthesitis and dactylitis and the greatest HAQ-DI change and had a higher rate of achieving combined ACR 50 and PASI 100 response than TNFi, and patients receiving PDE4i were least likely to have adverse events. Taking into account both efficacy and safety, IL-17Ai may be the better biological agent for PsA treatment. These findings may facilitate understating of different categories of biologics, and assist in clinical decision-making for the treatment of biological-naïve patients with PsA.

Many network meta-analyses have been conducted to explore the efficacy and safety of specific biologics in PsA over the past 20 years or so (Migliore et al., 2021). Recently, infliximab, guselkumab, adalimumab, golimumab, secukinumab, and ustekinumab were shown by a network meta-analysis to be possibly safer and more effective than other targeted DMARDs in induction therapy of active PsA (Lu et al., 2019). Ruyssen-Witrand et al. (2020) also compared specific biologics in PsA via a network meta-analysis, and illustrated that infliximab was superior to other biologics for ACR and PASI response. Another network meta-analysis focused on comparison of 14 small-molecule biologics for PsA patients (Qiu et al., 2020), and reported that golimumab, etanercept and infliximab could be the optimum agents in terms of efficacy and safety. These network meta-analyses incorporated both biological-naïve and biological-experienced patients, and used placebo as the common comparator. This network meta-analysis exclusively paid attention to PsA patients who were naïve to biologics and compare broad categories of biologics (TNFi, IL-17Ai, IL-12/23i, PDE4i, and JAKi) as regards efficacy and safety. Of note, we only included studies with head-to-head comparisons of these biologics since the evidence strength of direct comparison is greater than that of indirect comparison. Hence, studies with biologics versus placebo or methotrexate (MTX) alone were not included for the current analysis.

In terms of efficacy, the musculoskeletal endpoint (ACR 20/50/70, resolution of enthesitis, resolution of dactylitis), function endpoint (HAQ-DI change, ∆ HAQ-DI ≥ 0.35), and composite index endpoint (ACR 50 + PASI 100) were quantitatively assessed. JAKi were identified to have the highest likelihood of achieving ACR 20/50/70 response, followed by IL-17Ai in biological-naïve patients. JAKi are the latest drug class of disease-modifying medication to emerge for the treatment of rheumatoid arthritis (RA). Recent evidence has provided support for the effectiveness of JAKi regarding ACR 20/50/70 (Harkins et al., 2023; Lee and Song, 2023). The JAK family, including JAKi, JAK2, JAK3, and tyrosine kinase (TYK) 2, is related to signal transducers and activators of transcription (STAT) and serves as a crucial role in mediating downstream signaling of many important pro-inflammatory cytokines involved in the pathogenesis of PsA (Kvist-Hansen et al., 2020; Crispino and Ciccia, 2021). The biological agent JAKi cause suppression of the JAK/STAT pathway, and adjusts several inflammatory pathways via influencing various cytokines, which improve clinical manifestations, thus enhancing ACR response (Jamilloux et al., 2019; El Jammal et al., 2020). JAKi also exhibit rapid onset of action, role in reducing central pain, and impact on structural damage (Harrington et al., 2020). IL-17Ai were reported to inhibit disease activity associated with the skin, joints and entheses in spondyloarthritides including PsA (McGonagle et al., 2019), which may relate to the positive role of IL-17Ai in ACR 20/50/70 response. In addition, IL-17Ai were most likely to achieve the resolution of enthesitis and dactylitis, as shown in this paper, which suggested that IL-17Ai may be beneficial for relieving the musculoskeletal symptoms of PsA. As for the other key domains for PsA, peripheral arthritis, axial disease and skin and nail psoriasis, relevant information was missing in the included studies. A recent review showed that many therapeutic options, including IL-17Ai, IL-12/23i and JAKi, had similar effects on peripheral arthritis in patients with PsA (Ayan et al., 2023). Lubrano et al. (2023) reported that IL-17Ai and JAKi could be applied for axial disease treatment in PsA. Besides, among 14% of patients, peripheral arthritis, skin disease and nail psoriasis are the most common combination of PsA domains, and IL-17 inhibitors exhibited effectiveness across all domains (Mease et al., 2023). Future studies should pay more attention to these PsA domains and improve reporting of corresponding data. With respect to the function endpoint, patients receiving IL-17Ai were most likely to obtain the greatest HAQ-DI change, although no significant difference was found among involved IL-17Ai, TNFi and IL-12/23i biologics. According to previous reviews, ixekizumab relieved joint symptoms, improves function, and impede development of structural damage in PsA (Toussirot, 2018), and individuals with PsA had enhanced physical function and health-related quality of life after secukinumab usage (Blair, 2021). Secukinumab was well tolerated in general (Blair, 2021). For the endpoint ACR 50 + PASI 100 response considering both musculoskeletal and skin manifestations, IL-17Ai were superior to TNFi, indicating that IL-17Ai may have comprehensive control of PsA in biological-naïve patients. However, no other biologics were assessed in this endpoint, which necessitates future studies to investigate more biologics for simultaneous ACR 50 and PASI 100 response in PsA.

In terms of safety, PDE4i had the lowest probability to cause adverse events among the 5 kinds of biologics despite no significant difference observed among IL-17Ai, TNFi, JAKi, IL-12/23i and PDE4i biologics. PDE4i including apremilast blocks PDE4 enzyme and elevates the levels of intracellular cyclic adenosine monophosphate (cAMP), leading to downregulated inflammatory reactions through suppressing IL-17, interferon-γ, TNF, and so forth (Nassim et al., 2020; Picchianti-Diamanti et al., 2021), which may explain the potential adventage of PDE4i over other biologics. To be noted, merely apremilast was evaluated as a representative of PDE4i, the information of which was provided by one qualified study in the current network meta-analysis. Consistently, the safety of apremilast has been identified in existing research (Mease, 2014; Qu et al., 2016; Kang et al., 2022). The reporting of PDE4i′s efficacy in the future should also be improved for inclusion to facilitate comprehensive assessment. Concerning serious adverse events, McInnes et al. (2021) and Reich et al. (2022) reported the higher incidences of serious adverse events in patients receiving upadacitinib (30-mg dose) and adalimumab, respectively. According to European Medicine Agency (EMA) and US Food and Drug Administration (FDA) recent warnings, JAKi were associated with increased risks of major adverse cardiac events, cancer, venous thromboembolic events, severe infections, and death (Kragstrup et al., 2022; Philippoteaux et al., 2022), and clinicians needs to carefully consider risk factors and assess corresponding risks of individual patients before use of JAKi. For the arthritis activity endpoint, skin endpoint, and drug retention, qualitative descriptions were provided since unsynthesizable data from the included studies. Studies should adopt standardized reporting and a great number of investigations are required for these outcomes.

Based on the head-to-head evidence of different biologics for both efficacy and safety, IL-17Ai may be the most favorable treatment option to improve musculoskeletal, skin and function outcomes with few adverse effects for biological-naïve patients with PsA. Several limitations should be mentioned in interpreting the results. Firstly, the dosage and course of treatment of same biologics may vary in different included studies, possibly leading to increased heterogeneity between studies. Some data on TNFi were collected more than 15 years ago when PsA was less known and biologic treated disease severity was higher as compared to more recent trials, which may also increase heterogeneity. Besides, some patients in the included studies had comorbidities, such as diabetes, chronic obstructive pulmonary disease, asthma, emotional disorders, etc., which may affect the treatment results. Secondly, other key efficacy domains for PSA (axial disease, skin nails and related conditions) (Coates et al., 2022) were not covered by the current paper because no relevant data were provided by the included studies, which necessitates future studies to explore the effect of biologics on these outcomes. As shown in Table 1, only five studies reported the combination therapy with MTX alone (in some or all patients), two studies reported the combination therapy with MTX or other csDMARDs (data on MTX could not be distinguished from data on other csDMARDs), the remaining nine studies did not report relevant information on MTX. Thus, the clear separation of treatments with biologics combined with and not combined with MTX could not be achieved. Thirdly, some outcomes were evaluated by limited literature, and a small sample size may influence the stability of the results. There were relatively limited safety data and population sizes by biologics in the original papers, which requires more patients in a group and with long-term follow up to enrich safety data in future research. Additionally, some outcomes could only be qualitatively described. Finally, due to the lack of relevant information on sponsor-supported open-label extensions (OLEs) and non-sponsored OLEs, sponsor-supported OLEs and non-sponsored OLEs could not be split. Studies should improve their reporting of these aspects to promote deeper comprehensive research.

## Conclusion

In 2023, JAKi had the highest probability of achieving ACR 20/50/70 response, and IL-17Ai were most likely to realize the resolution of enthesitis and dactylitis and the greatest HAQ-DI change and had a higher rate of combined ACR 50 and PASI 100 response. Patients receiving PDE4i were least likely to have adverse events, despite no significant difference among different biologics. Considering both efficacy and safety, IL-17Ai may be the better option for biological-naïve patients with PsA requiring biological therapy. Future studies are warranted for validation.

## Data Availability

The original contributions presented in the study are included in the article/supplementary material, further inquiries can be directed to the corresponding author.
